# Peptide nucleic acids targeting β-globin mRNAs selectively inhibit hemoglobin production in murine erythroleukemia cells

**DOI:** 10.3892/ijmm.2014.2005

**Published:** 2014-11-14

**Authors:** GIULIA MONTAGNER, CHIARA GEMMO, ENRICA FABBRI, ALEX MANICARDI, IGEA ACCARDO, NICOLETTA BIANCHI, ALESSIA FINOTTI, GIULIA BREVEGLIERI, FRANCESCA SALVATORI, MONICA BORGATTI, ILARIA LAMPRONTI, ALBERTO BRESCIANI, SERGIO ALTAMURA, ROBERTO CORRADINI, ROBERTO GAMBARI

**Affiliations:** 1 Department of Life Sciences and Biotechnology, University of Ferrara, Ferrara, Italy; 2 Department of Chemistry, University of Parma, Parma, Italy; 3 IRBM Science Park, Pomezia (RM), Italy

**Keywords:** peptide nucleic acids, sickle-cell anemia, β-globin, hemoglobin, erythroid differentiation

## Abstract

In the treatment of hemoglobinopathies, amending altered hemoglobins and/or globins produced in excess is an important part of therapeutic strategies and the selective inhibition of globin production may be clinically beneficial. Therefore the development of drug-based methods for the selective inhibition of globin accumulation is required. In this study, we employed peptide nucleic acids (PNAs) to alter globin gene expression. The main conclusion of the present study was that PNAs designed to target adult murine β-globin mRNA inhibit hemoglobin accumulation and erythroid differentiation of murine erythroleukemia (MEL) cells with high efficiency and fair selectivity. No major effects were observed on cell proliferation. Our study supports the concept that PNAs may be used to target mRNAs that, similar to globin mRNAs, are expressed at very high levels in differentiating erythroid cells. Our data suggest that PNAs inhibit the excess production of globins involved in the pathophysiology of hemoglobinopathies.

## Introduction

The majority of the molecular biology based approaches developed for the experimental therapy of thalassemia and sickle-cell anemia (SCA) are based on the induction of defective globin (β-globin in β-thalassemia) (1–5) or (also in association with this strategy) the induction of fetal hemoglobin (HbF) (6–10). The *de novo* production of adult hemoglobin (HbA) can be achieved in β^0^-thalassemias by gene therapy (1–4) and gene correction, by homologous recombination (11,12) and/or by the treatment of erythroid cells with molecules causing read-through (13). The induction of HbF can be obtained by using low molecular weight drugs causing the induction of the γ-globin gene (6–8,14–17), artificial promoters (18,19), decoy molecules targeting transcription factors involved in the transcriptional repression of γ-globin genes (MYB, KLF-1 and BCL-11A) (20,21), or microRNAs targeting mRNAs coding for these repressors (data are available for microRNAs miR-15a, miR-16-1, miR-486-3p and miR-23a/27a) (22–24). On the other hand, clinical complications in SCA and β-thalassemia are also related to the production of defective proteins (β-globin in SCA) (25–27) or to the accumulation of free globins which are not organized in a functional tetramer (such as in the case of free α-globins in β-thalassemia) (28,29). It is well known that sickle hemoglobin (HbS) has peculiar biochemical properties, leading to polymerization when deoxygenated. HbS polymerization is associated with a reduction in cell ions and water content (cell dehydration) and increased red cell density, which further accelerates HbS polymerization. Pathophysiological studies have indicated that the dense, dehydrated red cells may play a central role in acute and chronic clinical manifestations of sickle-cell disease, in which intravascular sickling in capillaries and small vessels leads to vaso-occlusion and impaired blood flow in a variety of organs and tissues (30). Therefore, the development of drug-based methods for the inhibition of the accumulation of defective hemoglobins (HbS in SCA) or globin produced in excess (α-globins in β-thalassemia) is required (31,32).

In this field of investigation, peptide nucleic acids (PNAs) may be of great interest (33). PNAs are DNA analogues in which the sugarphosphate backbone is replaced by N-(2-aminoethyl)glycine units (33). These very interesting molecules were described for the first time by Nielsen *et al* (34) and, despite a radical structural change with respect to DNA and RNA, they are capable of sequence-specific and efficient hybridization with complementary DNA and RNA, forming Watson-Crick double helices (35). In addition, they are able to generate triple helix formation with double-stranded DNA and perform strand invasion (34). Accordingly, PNA-based analogues have been proposed as antisense molecules targeting mRNAs and microRNAs, triple-helix forming molecules targeting eukaryotic gene promoters, artificial promoters and decoy molecules targeting transcription factors (36). To the best of our knowledge, PNAs have not yet been employed to inhibit the expression of globin genes in erythroid cells. Thus, the aim of this study was to verify whether PNAs targeting globin mRNAs can be used to modulate globin gene expression and to reduce the level of a given type of globin. For this purpose, we produced one PNA targeting murine adult β-globin mRNAs and another recognizing the human γ-globin and β-globin mRNAs. These PNAs were tested on relevant target erythroid cell lines, such as the murine erythroleukemia (MEL) cell line. Erythroid differentiation and the high production of hemoglobins were induced by treatment with dimethylsulfoxide (DMSO) and hexamethylene bisacetamide (HMBA) (37–39).

## Materials and methods

### Synthesis and characterization of PNAs

The synthesis of the two PNAs was performed using standard automated Fmoc-based chemistry with HBTU/DIPEA coupling on a ChemMatrix resin loaded with Fmoc-Gly-OH as first monomer (loading 0.2 mmol/g, 5 μmol scale), on a Syro II peptide synthesizer, using commercially available monomers (Link Technologies, Bellshill, UK); Fmoc-Arg (Pbf)-OH (Sigma-Aldrich, St. Louis, MO, USA) was used for octaarginine synthesis. PNA purification was performed by reversed-phase high-performance liquid chromatography (RP-HPLC) with UV detection at 260 nm using a semi-prep column C18 (10 μm, 300×7.7 mm, Xterra Waters, 300 Å), eluting with water containing 0.1% TFA (eluent A) and acetonitrile containing 0.1% TFA (eluent B); elution gradient: from 100% A to 50% B in 30 min, flow: 4 ml/min. The purity and identity of the purified PNA were examined by ultra-performance liquid chromatography tandem mass-spectrometry (UPLC-MS; Waters Acquity equipped with ESI-Q analizer) using an Acquity UPLC BEH C18; 2.1×50 MM, 1.7 μm column. Anti-M-βglob-PNA: yield, 6%; electrospray ionization mass spectrometry (ESI-MS): m/z found (calculated): 1267.3 (1267.3) [MH_5_^5+^], 1056.2 (1056.2) [MH_6_^6+^], 905.4 (905.5) [MH_7_^7+^], 792.2 (792.4) [MH_8_^8+^], 704.5 (704.5) [MH_9_^9+^]; calculated MW: 6331.39. Anti-H-γglob-PNA: yield: 8%; ESI-MS: m/z found (calculated): 1346.0 (1345.4) [MH_4_^4+^]; 1076.8 (1076.5) [MH_5_^5+^], 897.7 (897.3) [MH_6_^6+^], 769.5 (769.2) [MH_7_^7+^], 673.3 (673.2) [MH_8_^8+^], 598.8 (598.5) [MH_9_^9+^], 539.0 (538.8) [MH_10_^10+^]; calculated MW: 5377.54.

### MEL and K562 cell lines and culture conditions

MEL cells (37–39) were cultured in humidified atmosphere of 5% CO_2_/air in RPMI-1640 medium (Sigma-Aldrich) supplemented with 10% fetal bovine serum (FBS; Biowest, Nuaillé, France), 50 U/ml penicillin and 50 μg/ml streptomycin (39). DMSO and HMBA were from Sigma-Aldrich. Stock solutions of HMBA were stored at −20°C in the dark and diluted immediately before use. Treatment of the MEL cells with HMBA and DMSO was carried out by adding the appropriate drug concentrations at the beginning of the cultures (30,000 cells/ml were seeded). To determine the effects of the treatments on the proliferation of the MEL cells, cell growth was monitored by determining the cell number/ml using a Z1 Coulter Counter (Coulter Electronics, Hialeah, FL, USA). Erythroid differentiated MEL cells containing hemoglobin were detected by specific reaction with a benzidine/hydrogen peroxide solution (0.2% in 5 M glacial acetic acid and 10% H_2_O_2_). The K562-D5 cell line was employed as it produces, in addition to hemoglobin (Hb) Gower1 and hemoglobin (Hb) Portland, HbF and HbA (40).

### RNA extraction

The cells were isolated by centrifugation at 1,500 rpm for 10 min at 4°C, washed with phosphate-buffered saline (PBS) and lysed with TRI-reagent™ (Sigma-Aldrich) according to the manufacturer’s instructions. The isolated RNA was washed once with cold 75% ethanol, dried and dissolved in nuclease-free pure water prior to use.

### Quantitative reverse transcription polymerase chain reaction (RT-qPCR)

For gene expression analysis, 500 ng of total RNA were reverse transcribed using random hexamers. Quantitative PCR assays were carried out using gene-specific double-quenched probes containing a 5′-FAM fluorophore, a 3′-IBFQ quencher and an internal ZEN quencher. The nucleotide sequences used for the RT-qPCR analysis of mouse globin mRNAs were α-globin forward, 5′-CTG ACC TCC AAG TAC CGT TAA G-3′ and reverse primer, 5′-GCT TCT TCC TAC TCA GGC TTT AT-3′ and α-globin probe, 5′-/56-FAM/TCT CTC CCT/ZEN/TGC ACC TGT ACC TCT/3IABkFQ/-3′; β-globin forward, 5′-GGA AAG GTG AAC TCC GAT GAA-3′ and reverse primer, 5′-TGA TAG CAG AGG CAG AGG ATA G-3′ and β-globin probe, 5′-/56-FAM/CCT TGG ACC/ZEN/CAG CGG TAC TTT GAT/3IABkFQ/-3′. The primers and probes used to assay mouse globins were purchased from Integrated DNA Technologies (IDT; Coralville, IA, USA). The relative expression was calculated using the comparative cycle threshold method and the endogenous control mouse gene, GAPDH, was used as a reference gene (PrimeTime Mm.PT.39a.1, IDT).

### HPLC analysis

The cells were harvested, washed once with PBS and the pellets were lysed in lysis buffer (sodium dodecyl sulphate 0.01%). After spinning for 1 min in a microcentrifuge, the supernatant was collected and stored at 4°C. Hemoglobins in the lysates were separated by cation-exchange HPLC (Pharmacia LKB Gradient Pump 2249, VWM 2141), using a Synchropak CM300 (250×4.6 mm) column (Eichrom Technologies, Inc., Darien, IL, USA) and BisTris (30 mM) buffer. Standard HbA and HbF (Sigma-Aldrich, Milwaukee, WI, USA; Helena Laboratories, Beaumont, TX, USA) solutions were used as a reference.

### Bioinformatics analysis

The secondary structure of the mouse β^major^- and β^minor^-globin mRNA sequences, 5′ untranslated region (UTR), coding sequence (CDS) and 3′UTR, was predicted using the program available online TBI ViennaRNA Web Services (http://rna.tbi.univie.ac.at). The mouse β-globin reference sequences analyzed were obtained from the NCBI website.

### Statistical analysis

The results are expressed as the means ± standard error of the mean (SEM). Comparisons between groups were made using a paired Student’s t-test and one-way analysis of variance (ANOVA). Statistical significance was defined with p<0.01.

## Results

### Design of PNAs

**Figure 1:**
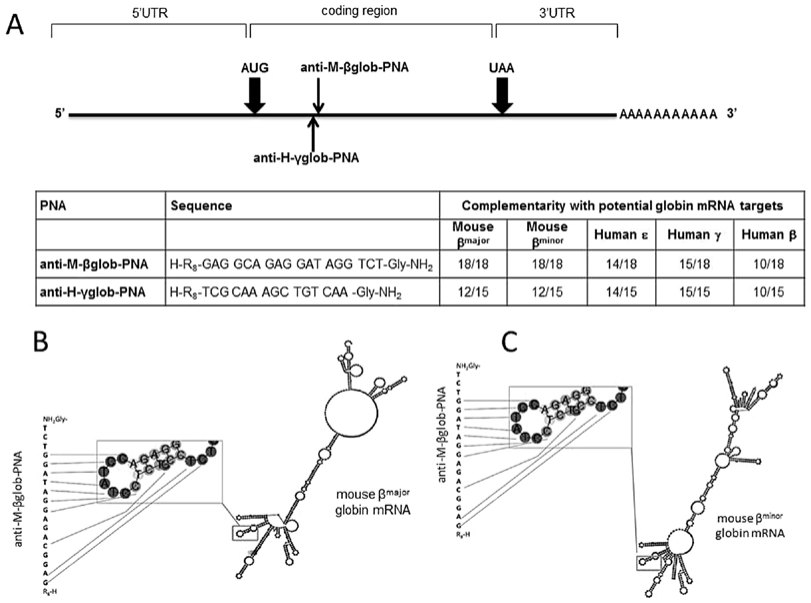
(A) Location of the binding sites (upper panel) and sequences of the PNAs employed in our study (lower panel). (B and C) Possible interactions between anti-M-βglob-PNA and mouse (B) β^major^ and (C) β^minor^ globin mRNAs. Predicted secondary structure of the 5′UTR, CDS and 3′UTR region of mouse β^major^- and β^minor^-globin mRNAs were based on the NCBI website references sequences, NM_001278161.1 and NM_016956.3, respectively and obtained using the TBI ViennaRNA Web Services (http://rna.tbi.univie.ac.at/). Magnification of the central portion of the CDS site of the globins points out the possible interaction between the β-globin CDS target strands and the anti-M-βglob-PNA region. PNA, peptide nucleic acid.

The location of the binding sites and the sequences of the PNAs employed in this study are presented in Fig. 1. As clearly appreciable from the analysis of the sequences recognized, the anti-M-βglob-PNA is able to hybridize to a region of both mouse β^major^- and β^minor^-globin mRNAs exhibiting similar predicted secondary structures (Fig. 1B and C). This feature allows us to study the effects of the PNAs simply analyzing the proportion of erythroid differentiated MEL cells.

These cells, upon the induction of erythroid differentiation with HMBA or DMSO produce almost exclusively Hb^major^ (α_2_β_2_^major^) and Hb^minor^ (α_2_β_2_^minor^). Moreover, these PNAs display 4 to 8 mismatches with human β-globin (8 mismatches), γ-globin (3 mismatches) and ɛ-globin (4 mismatches) (Fig. 1A, bottom panel). This allows us to verify possible non-specific inhibitory effects when used on K562 cell clones subjected to erythroid differentiation which produce, upon treatment with mithramycin (MTH), high levels of Hb Gower 1 (ζ_2_ɛ_2_), Hb Portland (ζ_2_γ_2_), HbF (α_2_γ_2_) and HbA (α_2_β_2_) (40). Conversely, as control PNA molecules we used anti-H-γglob-PNA recognizing the evolutionarily homologue human γ-globin mRNAs and displaying 3 mismatches with the murine β^major^ and β^minor^ mRNAs (Fig. 1A). Both anti-M-βglob-PNA and anti-H-γglob-PNA were linked with a R_8_ polyarginine peptide, since it has been reported in several studies that the uptake of PNAs by target cells is difficult (41,42). R_8_ was employed in order to maximize PNA uptake, as reported by our group for several PNAs, which, without exception, are internalized with very high efficiency to target cells (43–45).

### Effects of anti-M-βglob-PNA on the growth of MEL cells

**Figure 2:**
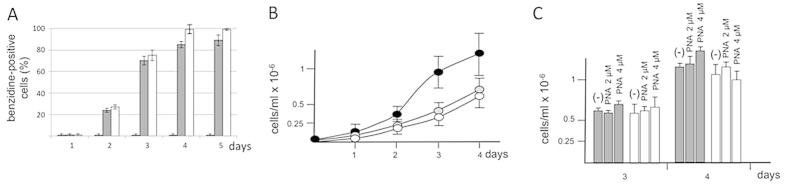
(A) Level of erythroid differentiation obtained when murine erythroleukemia (MEL) cells are treated with 2% dimethylsulfoxide (DMSO) (grey boxes), or with 2.5 mM hexamethylene bisacetamide (HMBA) (white boxes). Untreated cells, black boxes. (B) Proliferation of MEL cells cultured without inducers (black symbols), or in the presence of 2% DMSO (grey symbols), or 2.5 mM HMBA (white symbols). (C) Effects of 2 and 4 μM of anti-M-βglob-PNA on the proliferation of MEL cells treated with 2% DMSO (grey boxes), or with 2.5 mM HMBA (white boxes). PNA, peptide nucleic acid.

Fig. 2 shows the kinetics of differentiation (Fig. 2A) and cell growth (Fig. 2B) obtained when the MEL cells are treated with 2% and 2.5 mM DMSO and HMBA, respectively. The high level of induction (>80% after 3 or 4 days in all the experiments performed) confirms that this cellular system is excellent to determine inhibitors of the expression of adult β-globin genes, since, unlike other erythroid cellular model systems (such as human K562 cells), these cells mostly produce the adult-type Hb^major^ and Hb^minor^ hemoglobins (39). As shown in Fig. 2C, the addition of anti-M-βglob-PNA did not alter the proliferation rate of these cells, formally demonstrating no cytotoxic effects of this PNA on the MEL cells, either in the absence of differentiation inducers (data not shown) or in the presence of DMSO or HMBA.

### Anti-M-βglob-PNA inhibits the erythroid differentiation of MEL cells induced by DMSO and HMBA

**Figure 3:**
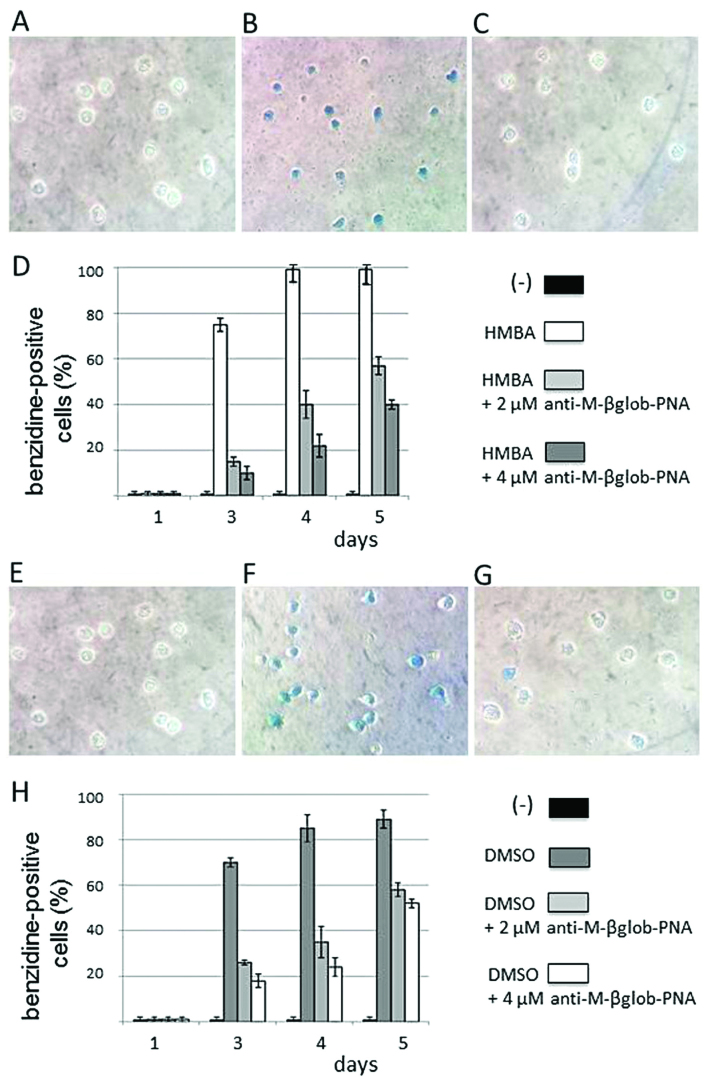
Effects of anti-M-βglob-PNA on erythroid differentiation of murine erythroleukemia (MEL) cells induced by (A–D) hexamethylene bisacetamide (HMBA) or (E–H) dimethylsulfoxide (DMSO). Intracellular accumulation of hemoglobin was determined by the benzidine staining of treated cells. (A–C and E–G) Representative examples of benzidine staining of (A and E) untreated cells, or cells treated with (B) HMBA, (C) HMBA plus 4 μM anti-M-βglob-PNA, (F) DMSO or (G) DMSO plus 4 μM anti-M-βglob-PNA. (D–H) Summary of 3 independent experiments performed with (D) HMBA or (H) DMSO without the addition of the PNA or in the presence of the indicated concentrations of anti-M-βglob-PNA. PNA, peptide nucleic acid.

The effects of anti-M-βglob-PNA on the erythroid differentiation of MEL cells were first analyzed by benzidine staining of the treated cells. The results of this experiment are presented in Fig. 3, which clearly illustrates that the MEL cells treated with DMSO (Fig. 3G and H) and HMBA (Fig. 3C and D) do not efficiently differentiate in the presence of anti-M-βglob-PNA. Fig. 3A–C and E–G shows representative experiments, while Fig. 3D and H shows the summary of 3 independent experiments, confirming a decrease in the proportion of benzidine-positive (hemoglobin-containing) PNA-treated cells.

### Effects of anti-M-βglob-PNA on hemoglobin and β-globin mRNA accumulation in MEL cells treated with HMBA

**Figure 4:**
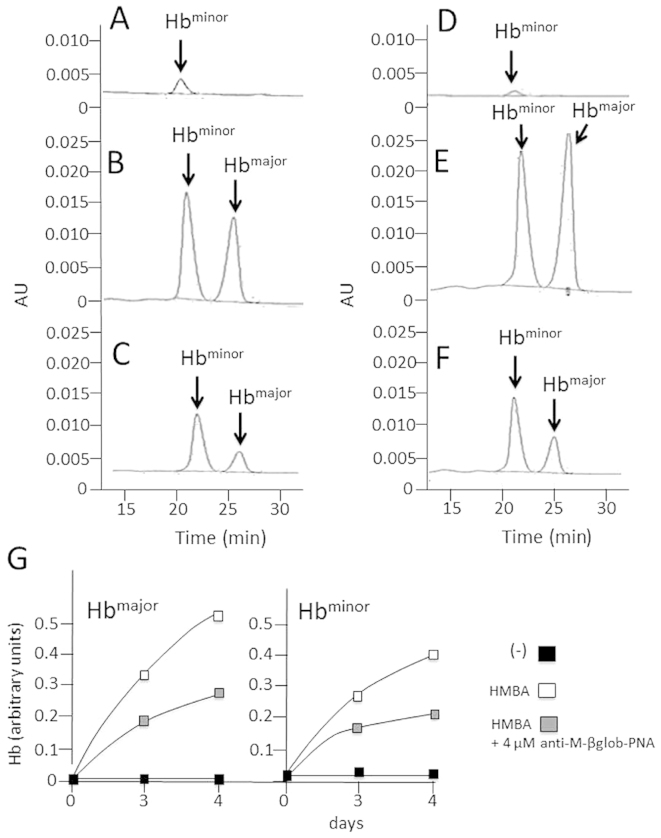
Effects of anti-M-βglob-PNA on hemoglobin accumulation. Representative HPLC analysis of (A and D) untreated murine erythroleukemia (MEL) cells, (B and E) hexamethylene bisacetamide (HMBA)-treated cells or (C and F) cells treated with HMBA in the presence of 4 μM anti-M-βglob-PNA; (A–C) 3 days of treatment and (D–F) 4 days of treatment. (G) Quantitative analyses of Hb^major^ and Hb^minor^. PNA, peptide nucleic acid.

The effects of anti-M-βglob-PNA on hemoglobin and β-globin mRNA accumulation were examined in the HMBA-treated cells by HPLC (for hemoglobin analysis) and RT-qPCR (for the analysis of β-globin mRNA). The results are presented in Fig. 4, which clearly illustrates that anti-M-βglob-PNA inhibited the accumulation of both Hb^major^ and Hb^minor^ hemoglobins fully in agreement with the data shown in Fig. 3. Of note, anti-M-βglob-PNA also inhibited, to a certain extent, β-globin mRNA, suggesting that the inhibition of hemoglobin production may be also associated with the lower stability of β-globin mRNA (data not shown).

### Specificity of the effects of anti-M-βglob-PNA

**Figure 5:**
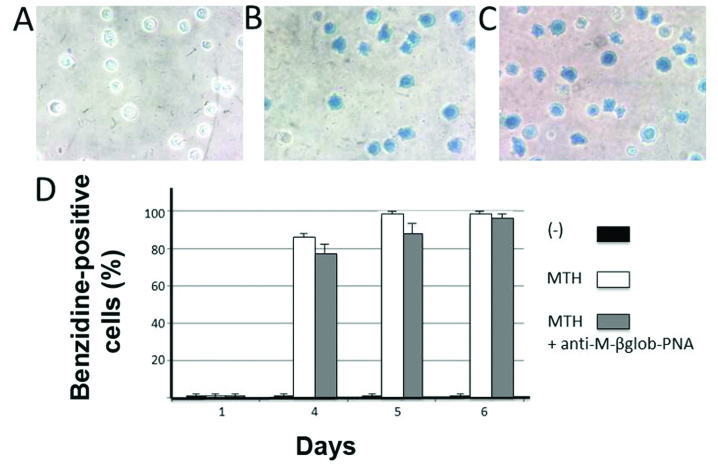
Lack of effects of anti-M-βglob-PNA on erythroid differentiation of K562-D5 cells induced by mithramycin (MTH). Intracellular accumulation of hemoglobin was determined by benzidine staining. (A–C) Representative examples of benzidine staining of (A) untreated K562-D5 cells, or cells treated for 4 days with (B) MTH or (C) MTH plus 4 μM anti-M-βglob-PNA. (D) Summary of 3 independent experiments. The proportion of benzidine-positive cells was determined at the beginning of the treatment (day 1) and after 4, 5 and 6 days of cell culture without treatment (black boxes), or treatment with MTH 20 nM in the absence (white box) or in the presence (grey boxes) of 4 μM anti-M-βglob-PNA. PNA, peptide nucleic acid.

**Figure 6:**
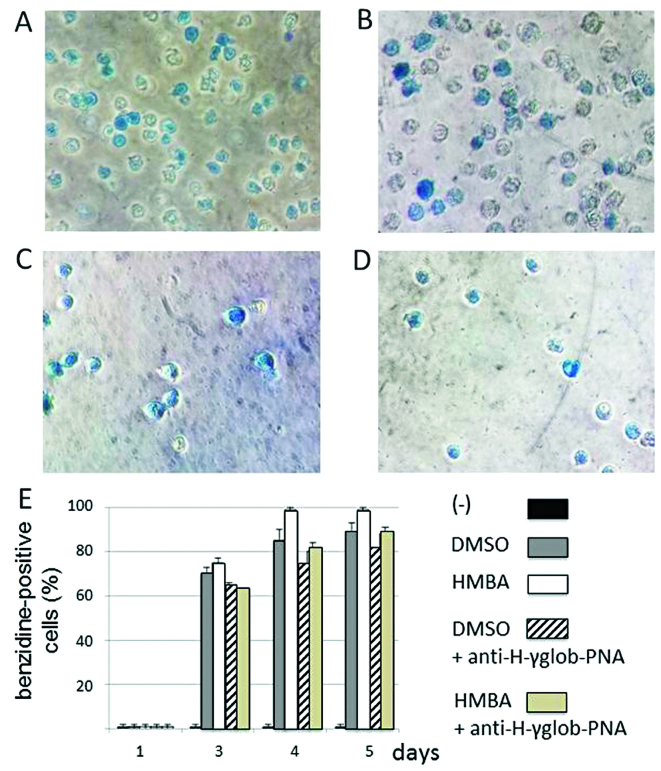
Lack of effects of anti-H-γglob-PNA on erythroid differentiation of murine erythroleukemia (MEL) cells induced by dimethylsulfoxide (DMSO) or hexamethylene bisacetamide (HMBA). Intracellular accumulation of hemoglobin was determined by benzidine staining. (A–D) Representative examples of benzidine staining of MEL cells treated for 3 days with (A and B) 2% DMSO or (C and D) 2.5 mM HMBA (A and C) in the absence or (B and D) in the presence of 4 μM anti-H-γglob-PNA. (E) Summary of 3 independent experiments. The proportion of benzidine-positive cells was determined at the beginning of the treatment (day 1) and after 3, 4 and 5 days of cell culture performed as described in the index. PNA, peptide nucleic acid.

The effects of anti-M-βglob-PNA on erythroid differentiation were also examined using human K562 cells subjected to erythroid differentiation by treatment with mithramycin (MTH). As shown in Fig. 5 no inhibitory effects of anti-M-βglob-PNA were observed on the MTH-stimulated K562-D5 cell clones, indicating high levels of specificity of the inhibitory effects of the anti-M-βglob-PNA. Furthermore, control experiments were also performed using the anti-H-γglob-PNA on HMBA- and DMSO-treated MEL cells. Of note, no inhibitory effects were observed using the anti-H-γglob-PNA, suggesting that the effects of treatment of the erythroid cells with PNAs were sequence-specific (Fig. 6).

## Discussion

In the treatment of SCA, HbS appears to be an important therapeutic target, since its polymerization is responsible for the sickling of SCA red-blood cells and important adverse clinical parameters (25–27). For instance, intravascular sickling in capillaries and small vessels leads to vaso-occlusion and impaired blood flow in a variety of organs and tissues (30). These conclusions are sustained by strong evidence suggesting that the induction of HbF following the treatment of SCA patients with hydroxyurea (HU) appears to be beneficial, due to the intrinsic anti-sickling properties of HbF (46,47). In any case, the inhibition of β-globin may be beneficial, allowing further reduction of sickling properties. Therefore the proof-of-principle of PNA-based methods enabling the effective inhibition of the accumulation of β-globin is of interest and of potential biomedical application.
